# Capillary Electrophoresis Mass Spectrometry for Scalable Single-Cell Proteomics

**DOI:** 10.3389/fchem.2022.863979

**Published:** 2022-04-08

**Authors:** Bowen Shen, Leena R. Pade, Sam B. Choi, Pablo Muñoz-LLancao, M. Chiara Manzini, Peter Nemes

**Affiliations:** ^1^ Department of Chemistry and Biochemistry, University of Maryland, College Park, MD, United States; ^2^ Pharmacology and Physiology, School of Medicine and Health Sciences, The George Washington University, Washington, DC, United States; ^3^ Department of Neuroscience and Cell Biology and Child Health Institute of New Jersey, Rutgers Robert Wood Johnson Medical School, The State University of New Jersey, New Brunswick, NJ, United States; ^4^ Department of Cell Biology, Yale University School of Medicine, New Haven, CT, United States

**Keywords:** capillary elechophoresis, mass spectrometry, single cell, xenopus, mouse, proteomics

## Abstract

Understanding the biochemistry of the cell requires measurement of all the molecules it produces. Single-cell proteomics recently became possible through advances in microanalytical sample preparation, separation by nano-flow liquid chromatography (nanoLC) and capillary electrophoresis (CE), and detection using electrospray ionization (ESI) high-resolution mass spectrometry (HRMS). Here, we demonstrate capillary microsampling CE-ESI-HRMS to be scalable to proteomics across broad cellular dimensions. This study established proof-of-principle using giant, ∼250-µm-diameter cells from embryos of the frog *Xenopus* *laevis* and small, ∼35-µm-diameter neurons in culture from the mouse hippocampus. From ∼18 ng, or ∼0.2% of the total cellular proteome, subcellular analysis of the ventral-animal midline (V11) and equatorial (V12) cells identified 1,133 different proteins in a 16-cell embryo. CE-HRMS achieved ∼20-times higher sensitivity and doubled the speed of instrumental measurements compared to nanoLC, the closest neighboring single-cell technology of choice. Microanalysis was scalable to 722 proteins groups from ∼5 ng of cellular protein digest from identified left dorsal-animal midline cell (D11), supporting sensitivity for smaller cells. Capillary microsampling enabled the isolation and transfer of individual neurons from the culture, identifying 37 proteins between three different cells. A total of 224 proteins were detected from 500 pg of neuronal protein digest, which estimates to a single neuron. Serial dilution returned 157 proteins from sample amounts estimating to about half a cell (250 pg protein) and 70 proteins from ca. a quarter of a neuron (125 pg protein), suggesting sufficient sensitivity for subcellular proteomics. CE-ESI-HRMS complements nanoLC proteomics with scalability, sensitivity, and speed across broad cellular dimensions.

## Introduction

Our understanding of cellular biochemistry depends on the ability to measure the constituent biomolecules in the cell. Genes and transcripts are now routinely analyzed with single-cell resolution thanks to automated sample handling using microfluidics, signal amplification using polymerase chain reaction (PCR), and multiplexing barcoding and sequencing using next-generation sequencing technologies. These measurements provided novel insights into regulatory mechanisms that control gene transcription as cells differentiate and establish lineages important for the formation of tissues and organs. Due to a complex correlation between transcription and translation, especially in developing biological systems, holistic understanding of cellular biochemistry calls for direct measurement of the proteome. Without a capability to amplify the whole proteome, the challenge has been due to limitations in the sensitivity of HRMS, the contemporary technology of choice for the unbiased and label-free identification and quantification of proteomes.

Single-cell HRMS was recently made possible for proteome studies. The technology was the focus of recent reviews (Rubakhin et al., 2011; Trouillon et al., 2013; Zenobi, 2013; Lombard-Banek et al., 2017; Levy et al., 2018; Zhang and Vertes, 2018; Kelly, 2020; Taylor et al., 2021). Advances in sample processing, separation, and detection enabled the measurement of proteins from minute amounts of materials that are available from cells. These studies usually tailored to specific types of cells in a given biological model. Using capillary microsampling, we demonstrated protein collection from identified cells in frog *X. laevis* and zebrafish embryos with dexterity, speed, and finesse. (Lombard-Banek et al., 2019; Lombard-Banek et al., 2021). We most recently extended capillary microsampling to single-neuron proteomics in tissues after electrophysiological recording of cellular activity. (Choi et al., 2022). Theoretically, capillary microsampling is scalable to varying cell dimensions.

NanoLC supports single-cell proteomics with automation and commercial availability. Systematic optimizations in sample handling and chromatography extended the technology to ultrasensitive proteomics with HRMS detection (Wilson et al., 2015; Duong et al., 2020; Kelly, 2020). The integrated proteome analysis device (iPAD) identified 635 proteins from 100 cells in an hour, raising a potential for high-throughput proteomics. (Chen et al., 2015). A miniaturized filter-aided sample preparation (Micro-FASP) approach enhanced sensitivity to 1,895 proteins from ∼200 ng protein digest in 50-cell *Xenopus* embryos. (Zhang et al., 2020). Data-independent acquisition identified a comparable number of proteins ( ∼1,600) from ∼40 ng of protein digest. (Saha-Shah et al., 2019). Peptide losses were reduced *via* automation and microscale reduction of the sample processing workflow, thus enhancing sensitivity. NanoPOTS downscaled sample processing to ∼200 nL, allowing ∼731 proteins to be identified from a single MCF10A cell. (Williams et al., 2020). About 1,000 proteins were detected from a single HeLa cell ( ∼200 pg protein content) using the approach with an ultra-low-flow nanoLC and a new-generational high-field asymmetric ion mobility spectrometer (Cong et al., 2021). These results forecast continuing developments in nanoLC-based single-cell HRMS proteomics.

CE is a viable and attractive alternative for single-cell proteomics. Single-cell CE was the focus of recent reviews (Sweedler and Arriaga, 2007; DeLaney et al., 2019; Stepanova and Kasicka, 2019; Nemes, 2021). An international study of 13 laboratories found CE-HRMS highly reproducible, supporting biological studies (Drouin et al., 2020). Sample amounts in CE compare favorably to those available from singe cells. Electrophoresis yields exquisite separation power to tackle complex peptidomes and can be integrated with ESI-HRMS. Our laboratory built microanalytical CE platforms to analyze ∼1–50 ng of single-cell proteomes from sample volumes limited to ∼100–250 nL. (Lombard-Banek et al., 2016a; Lombard-Banek et al., 2016b; Lombard-Banek et al., 2021). Diverse types of CE methods and designs of CE-ESI ion sources provided ever-increasing sensitivity [reviewed in ref (DeLaney et al., 2019)]. A third-generation electrokinetically pumped ultralow-flow CE-nanoESI interface extended analyses to femtograms of protein from cultured cells (Sun et al., 2013). This platform enabled the identification of ∼1,000 proteins from ∼50 ng of *Xenopus* protein digest on a quadrupole orbitrap mass spectrometer (Q Exactive HF). (Zhang et al., 2016). Our tapered-tip CE-ESI design yielded ∼1 pg (∼15 amol) of sensitivity for model proteins and ∼260 zmol (∼150,000 copies) for model peptides. (Choi et al., 2017). These custom-built CE-HRMS platforms identified ∼750 proteins from ∼5 ng of protein digest, or ∼0.05% of the total protein content of a single cell in a 16-cell stage frog embryo. (Lombard-Banek et al., 2019). The method returned 428 proteins from ∼500 pg of protein digest from cultured neurons, which estimated to single neurons. (Choi et al., 2021). Using commercial CE-ESI-MS, diluted protein digests of HeLa yielded 744 different proteins from single-cell equivalent protein amounts. (Johnson et al., 2022). In theory, CE-HRMS raises a possibility for scalable proteomics across cells of broad dimensions.

In a systematic study here, we assess CE-HRMS for scalable single-cell proteomics from large to small cells. For testing proof-of-principle, we capillary microsample protein content of ∼350 µm diameter identified embryonic cells (blastomeres) from 16-cell *X. laevis* embryos and single ∼35 µm diameter neurons cultured from the mouse. To improve sensitivity and analytical speed, we compare protein identifications from diluted protein digests from cultured neurons between nanoLC, the gold standard technology in bottom-up proteomics, and microanalytical CE using HRMS. Encouraged by the identification of hundreds of proteins from the embryonic cells and diluted protein digests estimating to single neurons or less, we also evaluate the platform for proteomics in single neurons in culture. Results from this study demonstrate CE-ESI-HRMS to complement nanoLC proteomics with scalability across broad cellular dimensions and a capability for subcellular sensitivity.

## Methods

### Chemicals and Materials

All solvents including acetic acid (AcOH), acetonitrile (ACN), formic acid (FA), methanol (MeOH), and water were purchased at LC-MS grade from Fisher Scientific. Reagents for bottom-up proteomics were purchased from Sigma-Aldrich (St. Louis, MO), unless specified otherwise. Ethylenediaminetetraacetic acid (EDTA) for neuron cell lysis was from Fisher Scientific (Fair Lawn, NJ). The CE *background electrolyte* (BGE) solution was prepared with 25% (v/v) ACN and 1 M FA. The CE-ESI *sheath solution* consisted of 10% (v/v) ACN containing 0.5% (v/v) AcOH. The capillaries for microsampling *Xenopus* single cells and performing nano-flow electrospray ionization (nanoESI) were constructed from borosilicate glass capillaries (0.50/1.00 and 0.75/1.00 mm inner/outer diameter, respectively) using a Sutter Instrument P-1000 micropipette puller (Novato, CA). The extracted proteins were digested with MS-grade TPCK-modified trypsin (part no. 90057, Pierce, Rockford, IL) in 200/500 µL LoBind Eppendorf vials (part no. 951010006/022431064, Eppendorf, USA), respectively, chosen to reduce adsorptive losses for proteins during sample processing. For CE, fused silica capillaries were purchased from Polymicro Technologies and used without modification (40/105 μm and 20/90 μm inner/outer diameter for *Xenopus* and mouse neurons, respectively, Phoenix, AZ). Measurements on the *Xenopus* digests used an electrokinetic pump to supply a low flow of sheath liquid, which was built using the pulled borosilicate capillary (Lombard-Banek et al., 2019). Analyses on the neuronal proteome digests employed a stainless steel tapered-tip metal emitter (100/320 μm inner/outer diameter, New Objective, Woburn, MA) following our recent design (Choi et al., 2018a).

### Animal Care

Adult *Xenopus laevis* frogs (Nasco, Fort Atkinson, WI) and pregnant C57BL/6 dams of mice (Charles River Laboratories, Wilmington, MA) were cared for and humanely used following protocols approved by the Institutional Animal Care and Use Committee of the University of Maryland, College Park (Approval no. #R-FEB-21-07) and the George Washington University (Approval no. A274), respectively.

### Protein Preparation From *Xenopus laevis* Cells

*Xenopus* embryos were collected during the natural mating of multiple parents. The experiments spanned different days and different parents to account for natural biological variability. The embryos were dejellied with 2% (w/v) cysteine solution and transferred to 100% (w/v) Steinberg’s solution (Lombard-Banek et al., 2019). The 2-cell stage embryos with stereotypical pigmentation were selected, cultured to the 16-cell stage, and the cell types of interest were recognized based on the location, pigmentation, and size. (Moody, 1987). The cell contents were aspirated by capillary microsampling following our recent study, (Lombard-Banek et al., 2019), then transferred to 50 mM ammonium bicarbonate (AmBic) prepared with HPLC grade water.

The extracted proteome was processed *via* standard bottom-up proteomic workflows. For the CE-MS measurements, traditional bottom-up proteomics steps such as reduction, alkylation, and desalting were eliminated to reduce material losses on the surfaces of the vial. The proteins were denatured at 60°C for 20 min, followed by digestion for 5 h at 37°C using trypsin (1 µL of 0.75 μg/μL for 18 ng of proteome, and 1 µL of 0.25 μg/μL for 5 ng of proteome). (Lombard-Banek et al., 2021). For the LC-MS measurements, the proteome was similarly processed, including reduction and alkylation, and desalted on a ZipTip column (Millipore Sigma) prior to analysis.

### Protein Preparation From Cultured Mouse Neurons

Primary cultures of isolated mouse hippocampal neurons were prepared as described elsewhere (Kaech and Banker, 2006). After 14 days of culture, the individual neurons were randomly selected in the culture for analysis. Alternatively, to process the entire cell population, the neurons were rinsed 3 times with 1 mL of sodium dodecyl sulfate (SDS) (10% v/v), then transferred into a LoBind microtube. The collected neurons were pelleted at 800 × *g* at 4°C for 5 min. The neurons were lysed in SDS with sonication, followed by reduction (5 μL of 1 M dithiothreitol, DTT, 30 min at 60°C), alkylation (10 μL of 1 M iodoacetamide, IAD, 15 min in the dark), and quenching of the reaction (5 μL of DTT). The extracted proteins were purified by acetone precipitation overnight, then reconstituted in 200 µL of 50 mM AmBic, followed by trypsin digestion (1 mg/mL, 0.8 µL) over 12 h at 37°C. The peptides were dried and reconstituted for analysis.

### NanoLC-HRMS

The peptides were dried at 60°C in a vacuum concentrator and reconstituted in LC-MS grade water containing 0.1% (v/v) FA. The peptide digest (400 ng) was trapped on a C18 trap column (100 μm i.d., 5 μm particle with 100 Å pores, 2 cm length, Acclaim PepMap 100, Thermo) at 5 μL/min for 5 min, then separated on a C18 analytical column (75 μm i.d., 3 μm particle with 100 Å pores, 25 cm length, Acclaim PepMap 100, Thermo) at 300 nL/min using a 120 min gradient using nanoflow liquid chromatography (Dionex Ultimate 3000 RSLC, Thermo). Elution employed the following gradient of Buffer B (100% ACN, 0.1% v/v FA) as follows (% v/v): 3.2% for 0–5 min, to 35% in 80 min, to 72% in 5 min, held at 72% for 5 min, then ramped to 5% in 5 min and equilibrated at 5% for 20 min. The eluent was electrosprayed through an electrified (+2 kV *vs*. Earth ground) stainless-steel emitter (part no. ES542, Thermo Fisher).

The generated ions were detected on an orbitrap-quadrupole-ion trap tribrid ultrahigh-resolution mass spectrometer (Orbitrap Fusion Lumos, Thermo) executing data-dependent acquisition (DDA) to control tandem MS using the following parameters: MS scan resolution, 120,000 FWHM (*m/z* 200) in the orbitrap analyzer; cycle time, 3 s; max IT, 50 ms; AGC target, 4 × 10^5^ counts; microscans, 1; dynamic exclusion, 60 s. The precursor ions were fragmented in the ion trap with the following settings: isolation window, 1.6 Da; higher-energy collisional dissociation (HCD) using the settings: normalized collision energy (NCE), 30%; AGC target, 1 × 10^4^ counts; max IT, 100 ms; microscans, 1.

### CE-ESI-MS

*Xenopus*. The peptide mixture was dried in a vacuum concentrator at 60°C and reconstituted in 2 µL of 75% (v/v) ACN containing 0.05% (v/v) FA (*sample solvent*). The peptides were separated in a custom-built CE-ESI platform, as described elsewhere. (Lombard-Banek et al., 2019; Lombard-Banek et al., 2021). Approximately 18 or 5 ng of peptides were hydrodynamically injected into a ∼90 cm CE capillary filled with the BGE. The sample was electrophoresed by electrifying the inlet end of the CE capillary, generating ∼5–10 µA current. An electrokinetic pump supplied low sheath-flow at the outlet of the CE capillary. The tip of the ESI emitter was mounted ∼1 mm from the mass spectrometer orifice. Approximately 1,000 V ESI voltage was applied to maintain a stable Taylor cone, (Nemes et al., 2007), monitored by a long-working distance objective microscope (Mitutoyo Plan Apo, Edmund Optics, Barrington, NJ) equipped with a CCD camera (EO-2018C, Edmund Optics). For 18 ng of *Xenopus* proteome digest, +23 kV CE voltage was applied, yielding ∼1 h separation time. For 5 ng of proteome digest, a +28 kV CE voltage reduced the analysis time to ∼30 min.

The separated peptides were measured on a quadrupole orbitrap mass spectrometer (Q Exactive Plus, Thermo Scientific) executing tandem MS using DDA. Dynamic exclusion on the 10 most abundant precursor ions were selected for fragmentation. The following MS (Rubakhin et al., 2011) parameters were used: scan range, *m/z* 350–900; mass resolution, 70,000 FWHM (*m/z* 200); AGC target, 1 × 10^6^ ions; C-trap maximum injection time, 50 ms. The MS (Levy et al., 2018) parameters were set as follows: isolation window for precursor ions, 1.5 Da; mass resolution, 17,500 FWHM (*m/z* 200); AGC target, 5 × 10^4^ ions; C-trap maximum injection time, 100 ms; HCD with nitrogen gas, 28% NCE.

### Neurons

The proteome digest from the individual neurons and neuron culture was dried in a vacuum concentrator at 60°C and respectively reconstituted in 0.5 and 5 µL of 50% (v/v) ACN containing 0.05% (v/v) AcOH (sample solvent). The CE *background electrolyte* (BGE) solution was 25% (v/v) ACN with 1 M FA. The CE-ESI *sheath solution* consisted of 0.1% FA and 50% (v/v) MeOH. The neuron samples were measured on a custom-built microanalytical CE-ESI platform previously developed in our group. (Choi et al., 2017). The peptides were separated in a 90 cm CE capillary and the CE voltage was set at 23 kV on the inlet of the capillary. The sheath solution was supplied at 300 nL/min and a stable Taylor cone was generated after applying +2,700 V ESI voltage. The tapered-tip emitter was set ∼2 mm from the MS orifice. The separated peptides were measured on the Q Exactive Plus mass spectrometer as earlier using DDA on the 15 most abundant precursor ions (Top 15) for MS/MS. The MS (Rubakhin et al., 2011) parameters were as follow: scan range, *m/z* 350–1,800; mass resolution, 35,000 FWHM (*m/z* 200); isolation window, *m/z* 0.8; C-trap maximum injection time, 50 ms. For MS (Levy et al., 2018), the parameters were set as follows: mass resolution, 17,500 FWHM (*m/z* 200), AGC target, 1.5 × 10^3^ ions; maximum injection time, 50 ms; HCD, nitrogen gas at 32% NCE.

### Data Analysis

The primary MS-MS/MS files were analyzed with Proteome Discoverer version 2.2 (Thermo) with SEQUEST search engine and Minora algorithm for label free quantification (LFQ) analysis or MaxQuant version 1.6.17.0 (Max Planck Institute of Biochemistry) executing the Andromeda search engine (Cox et al., 2014). Using identical search parameters between these bioinformatic platforms, the detected signals were identified against the *Xenopus laevis* proteome (downloaded from UniProt on 12 Sep 2021, containing 43,236 entries) or the mouse proteome (downloaded from SwissProt on 11 August 2021, containing 21,978 entries). The search parameters were as follows: minimum peptide length, 6 amino acids; maximal missed cleavage sites, 2; precursor mass tolerance, 10 ppm; fragment mass tolerance, 0.02 Da (Q Exactive Plus) and 0.6 Da (Lumos, Thermo). The static and dynamic modifications of cysteine carbamidomethylation and methionine oxidation were enabled for neuroproteomics by CE-MS and *Xenopus* proteomics by LC-MS. Protein identifications were filtered to <1% false discovery rate (FDR) against a reversed-sequence decoy database, both at the peptide and protein level. Common contaminant proteins were manually curated against the cRAPome (https://www.thegpm.org/crap/) and excluded from the proteins that are reported in this study.

### Safety

Standard safety protocols were followed when working with chemicals and biological samples. Care was taken when handling microsampling capillaries, electrospray emitters, and metal tapered-tip emitters, which pose needle-stick hazards. All electrically conductive parts of the CE-nanoESI platform were grounded or shielded to prevent electrical shock hazards.

### Data Repository

All primaryand processed MS-MS/MS data were deposited to the ProteomeExchange Consortium (http://proteomecentral.proteomexchange.org) *via* the PRIDE (Perez-Riverol et al., 2019) partner repository with the project accession number PXD031955.

## Results and Discussion

### CE-HRMS for Scalable Single-Cell Proteomics

**FIGURE 1:**
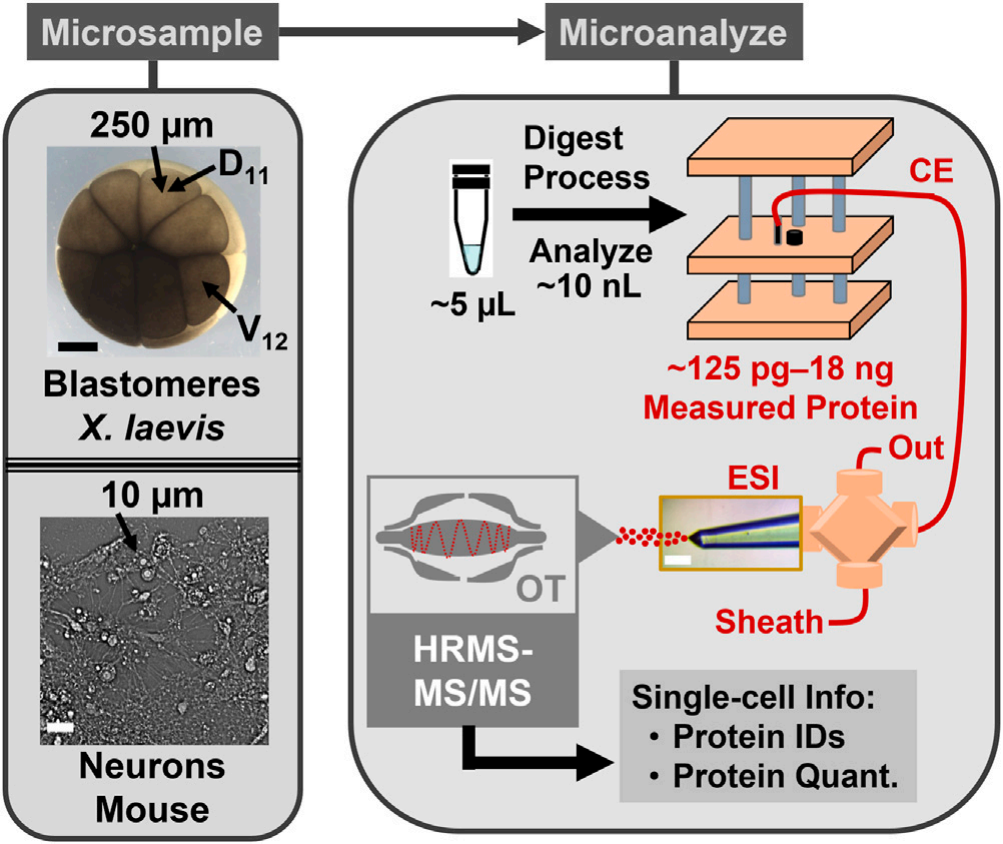
CE-HRMS for scalable single-cell proteomics. The protein content was collected from identified cells in *Xenopus laevis* embryos and neurons cultured from the mouse hippocampus. The example labels the normally neural-tissue fated dorsal-animal midline cell (D_11_) and the normally epidermally fated ventral-animal equatorial cell (V_12_). The proteins were detected on a custom-built microanalytical capillary electrophoresis (CE) electrospray ionization (ESI) platform using orbitrap (OT) high-resolution tandem mass spectrometry (HRMS-MS/MS).

The goal of this study was to assess the scalability of CE-HRMS for single-cell proteomics across broad cellular dimensions. Figure 1 presents the microanalytical pillars of our approach. Capillary microsampling was selected to enable the collection of proteins from cells of varying dimensions. We analyzed ∼30–300 µm (diameter) blastomeres from popular biological models in cell and developmental and neurobiology in this study. Early *X. laevis* embryos were used to obtain large, ∼250-µm-diameter embryonic cells that are fated to give rise to neural or epidermal tissues in the frog. The proteomic measurements were extended to differentiated cells, specifically ∼35 µm neurons in culture from the mouse. For the proteomic characterization of these cells, we benchmarked the performance of microanalytical CE using nanoLC as the closest neighboring reference technology and tested detection to cellular and subcellular amounts of proteins.

**FIGURE 2:**
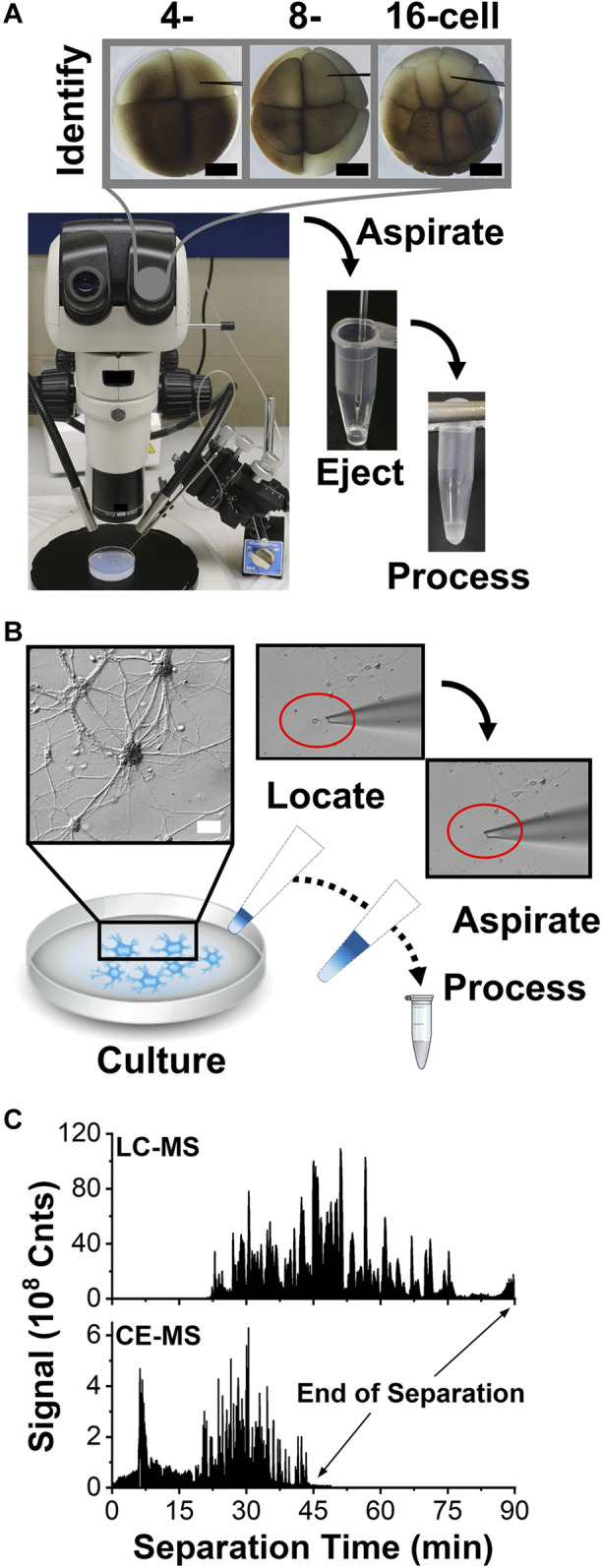
Scalable collection of trace amounts of proteins from single cells for HRMS detection. **(A)** Example showing microaspiration of cell contents into a microfabricated glass pipette from a D, D1, and D11 blastomere in a 4, 8-, and 16-cell *X. laevis* embryo. Scale, 250 µm. **(B)** Collection of a single cultured mouse neuron into a fabricated microcapillary. Scale, 20 µm. **(C)** Representative base-peak ion signal traces recorded between *m/z* 350–1,500 from 400 ng of digest using nanoLC and 10 ng of protein digest using CE, revealing enhancement in sensitivity and speed by electrophoresis.

Scalability was essential for these proteomic measurements. Figure 1 overviews our approach, starting with the identification of the cells of interest. The cleavage-stage *X. laevis* embryos provided an ideal biological model, because asymmetric pigmentation and stereotypical cell fate maps enabled accurate cell phenotyping and a large size ( ∼250 µm/cell in the 16-cell embryo) with predictable cell divisions facilitated direct manipulation of the cells. (Grant et al., 2013; Pratt and Khakhalin, 2013). Figure 2A demonstrates microscopy-aided capillary microsampling of identified cells in the embryos. The dorsal (called D) cell in the 4-cell embryo formed the dorsal-animal (called D1) cell of the 8-cell embryo, which then divided into the dorsal-animal midline (called D11) in the 16-cell embryo. Capillary microsampling ensured adequate spatial and temporal finesse and scalability to access contents of the cells in the complex embryo, despite synchronous cell divisions occurring every ∼15–30 min at this stage of development. An ∼10 nL portion was aspirated from the D11 cells into a pulled borosilicate capillary within ∼5 s using an in-line connected microinjector delivering negative pressure pulses. The aspirates were ejected into 5 µL of 50 mM of AmBic. This cell stores ∼8 µg of total protein within an ∼90 nL internal volume (Peuchen et al., 2016). Ca. 90% of the cellular proteome is from yolk, thus yielding an estimated 1 µg of yolk-free protein amount from each cell (Sun et al., 2016).

Microsampling was potentially applicable to also handling single mammalian neurons. To test the approach, we cultured primary hippocampal neurons on a glass slide. Figure 2B exemplifies readily identifiable neurons and their processes under inspection by an inverted microscope. A borosilicate tubing was fabricated into micropipettes with ∼30 µm tip diameter and mounted on a three-axis translation stage under remote control. As earlier, the outlet end of the micropipette was connected to a microinjector delivering pulses of calibrated vacuum. This micropipette was used to extract the whole neuron from the culture, including its soma and the microscopically identifiable portions of its axon and dendrites. Partial cell lysis was observed as the neuron was collected into the micropipette (see inset). We previously estimated that a single neuron yields ∼500 pg of total protein content. (Choi et al., 2018a). To account for smaller protein amounts than from the *X. laevis* cells, the neuron was captured together with ∼2 µL of Neurobasal Plus/B27 culture media to reduce protein losses, albeit added salts from the media were anticipated to challenge detection. The procedure was repeated on up to 3 different neurons in the culture.

The collected proteins were processed for bottom-up proteomics. The aspirates were ejected into a microtube containing AmBic and digested to peptides using trypsin following our established protocols (Lombard-Banek et al., 2019) (see Methods). The standard alkylation and reduction steps of bottom of proteomics were eliminated to reduce material losses for the neuron samples. The resulting peptides were separated over ∼2 h by gradient C18 reversed-phase chromatography or ∼30–60 min electrophoresis using CE. NanoLC analysis required at least ∼4 µL of protein digest in the sample loading vial, whence ∼2 µL of sample solution containing ∼400 ng of protein digest were injected during each technical replicate analysis. In comparison, our custom-built CE platform enabled the loading of ∼1–10 nL of the sample, or ∼1–10 ng of protein digest, from each ∼250 nL aliquot of the sample. These measured protein amounts mark at least an order of magnitude of improvement to help analyze lower protein amounts.

The resulting peptides were detected by ESI-HRMS. Figure 2C shows a representative base peak chromatogram and electropherogram from the same identified D11 *X. laevis* cell. Separation was completed in ∼90 min by LC and ∼45 min by CE; electrophoresis essentially halved the analysis time. The resulting peptide data allowed us to identify the source proteins against the *X. laevis* and mouse proteomes and estimate their relative abundances using established bioinformatic resources (<1% FDR), thus revealing the proteomic profile of the cells.

### Benchmarking CE Proteomics Using NanoLC

**FIGURE 3:**
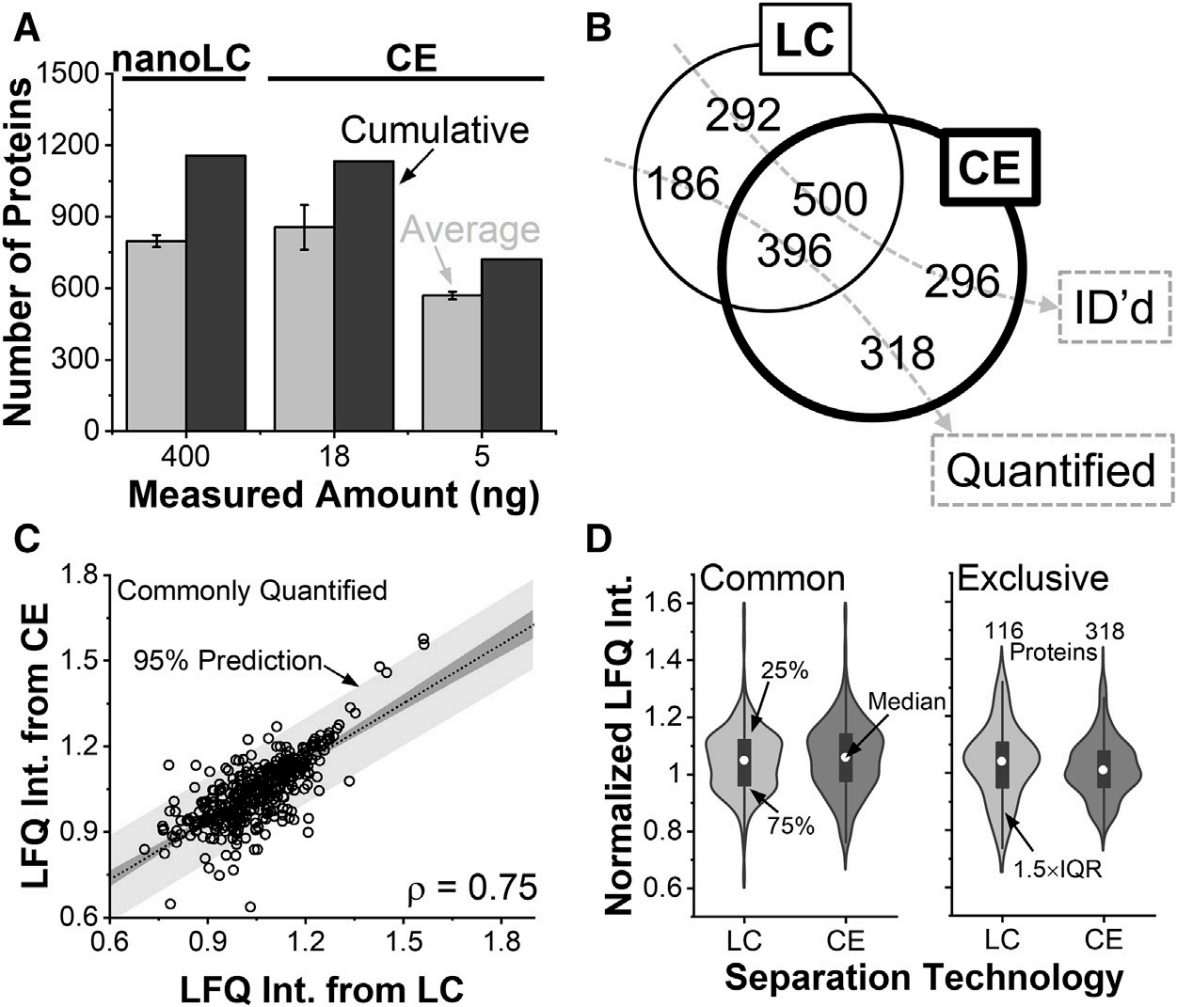
Benchmarking the analytical figures of merit. **(A)** Protein identifications in the V12 *X. laevis* cell from ∼400 ng of protein digest by nanoLC and ∼18 ng of protein digest by CE HRMS. **(B)** Single-shot analyses identifying (ID) complementary types of proteins. The CE experiment quantified more proteins than nanoLC. **(C)** Pearson correlation analysis of proteins that were quantifiable by both technologies. **(D)** Statistical comparison of protein quantification (Wilcoxon signed tests) revealing indistinguishable abundance distribution for commonly quantified proteins. Proteins that were only quantifiable by CE were detected in statistically higher abundance.

The proteomic data allowed for the evaluation of CE sensitivity to nanoLC, the closest neighboring single-cell technology. The content of a whole single V12 cell was capillary microaspirated and analyzed by both technologies as described earlier. Figure 3A compares the protein identifications from the ∼400 ng of protein digest by nanoLC and ∼18 ng protein digest by CE. Over ∼2 h of chromatography, a total of 797 proteins were identified on average, or 1,156 proteins in cumulative from technical triplicates. The proteins are tabulated in Table 1 in the electronic Supplementary Information (Supplementary Table S1).

We queried the reproducibility of the identifications. Using nanoLC, about 42% of the proteins were detected in all the replicates, and 65% were in at least two. Electrophoresis returned an average of 855, or 1,133 cumulative proteins from technical triplicates (see Supplementary Table S2). About 54% of the proteins were detected in all the replicates, and 73% were in at least two. From 5 ng of protein digest, the approach was scalable to an average of 569, or 722 cumulative proteins (See Supplementary Table S3). Therefore, identifications scaled better than anticipated based on protein amounts. We ascribed these improvements to field-amplified sample stacking allowing to concentrate the sample in the capillary for signal enhancement, higher theoretical plate numbers aiding peptide separation to tailor to the duty cycle of tandem MS, and reduced flow rates during nanoESI facilitating ionization efficiency. CE demonstrated favorable scalability toward trace amounts of protein digests.

The depth of protein identifications matter for biological experiments. Figure 3B compares the types of proteins that were identifiable a single shot measurement of the V12 blastomere using nanoLC (∼400 ng) and CE (∼18 ng). Despite analyzing a notably lower amount of proteome, ∼46% of all the proteins were identified by both technologies. A comparable number of proteins were exclusively identifiable by CE (296 proteins) and nanoLC (292 proteins). These identifications are tabulated in Supplementary Table S4.

As not all identifiable proteins are quantifiable in bottom-up proteomics, Figure 3B also compared protein quantification. Of all the 900 proteins that were quantified by the technologies, only ∼44% were commonly quantified. CE was able to quantify about twice as many unique proteins compared to nanoLC. Therefore, while identification was comparable, quantification was found enhanced during electrophoresis.

Orthogonality was compared among the quantifications. Using LFQ, an effective proxy for concentration in both technologies, Figure 3C plots the mean-normalized and log-transformed abundance of proteins that were quantified commonly between nanoLC and CE HRMS. With a calculated product moment (ρ) of 0.75, Pearson cross-correlation analysis found the two technologies reproducible at relatively quantifying the proteome. Figure 3D compares the normalized abundances of the proteins. Proteins that were quantifiable by both CE and nanoLC had indistinguishable abundance distributions (Wilcoxon signed test). Surprisingly, the 318 proteins that were quantifiable only by CE were statistically more abundant than the 186 proteins that were exclusively measured by nanoLC. Further, CE-MS achieved a lower number of missing values (∼10% missing values) among the quantitative replicates compared to nanoLC (∼27% missing values). Therefore, CE improved the sensitivity and robustness of quantification.

### Scaling to Neurons and Subcellular Protein Amounts

We analyzed the single neurons that were aspirated from the cell culture (recall Figure 2B). CE-ESI-HRMS returned 16, 14, and 7 proteins from single neurons, totaling 37 different proteins among the biological replicates (Supplementary Table S5). These results were encouraging, although revealed notable variability in identification success and proteome coverage. Manual handling of the cells and their processing in microvials likely introduced variations in protein aspiration and enhanced adsorptive losses on vial and pipette surfaces. We anticipate that our approach can readily be integrated with recent advances enabling high-efficiency preparation and processing of microscale samples [e.g., NanoPOTS (Zhu et al., 2018), SCoPe2 (Specht et al., 2021)]. Capillary microsampling allowed us to extend CE-HRMS proteomics to single neurons in culture.

These results also suggested scalability to smaller cells and subcellular compartments. To establish proof of principle, we limited variations in sample processing and protein losses by measuring diluted protein digests from neuron cultures in this portion of the study. We previously estimated a single cell to yield ∼ 500 pg of extractable protein amount. (Choi et al., 2018b). To enhance the detectable portion of the neuroproteome, we sought to improve both sensitivity and peptide sequencing. The protein digests were suspended in 75% (v/v) of ACN containing 0.05% (v/v) FA to aid sensitivity by enabling field-amplified sample stacking (Lombard-Banek et al., 2016a; Choi et al., 2017; Baxi et al., 2018; Choi et al., 2018b; Lombard-Banek et al., 2019; Lombard-Banek et al., 2021). We recently found concentration-dependent fragmentation redundancy to be a leading cause of sensitivity loss during CE HRMS employing DDA-driven tandem MS. (Choi et al., 2021). This experience allowed us to define an ion signal abundance-dependent threshold, or a rung of a DDA ladder from our previous work, to seek identifications. To maximize the utilization efficiency of the DDA duty cycle, the 250 peptide-like ions with the most abundant ion signals were excluded from sequencing. The approach was tested for total protein amounts approximating to a single cell (1 nL of 500 pg/nL analyzed), a half cell (1 nL of 250 pg/nL), and a quarter of a cell (1 nL of 125 pg/nL).

**FIGURE 4:**
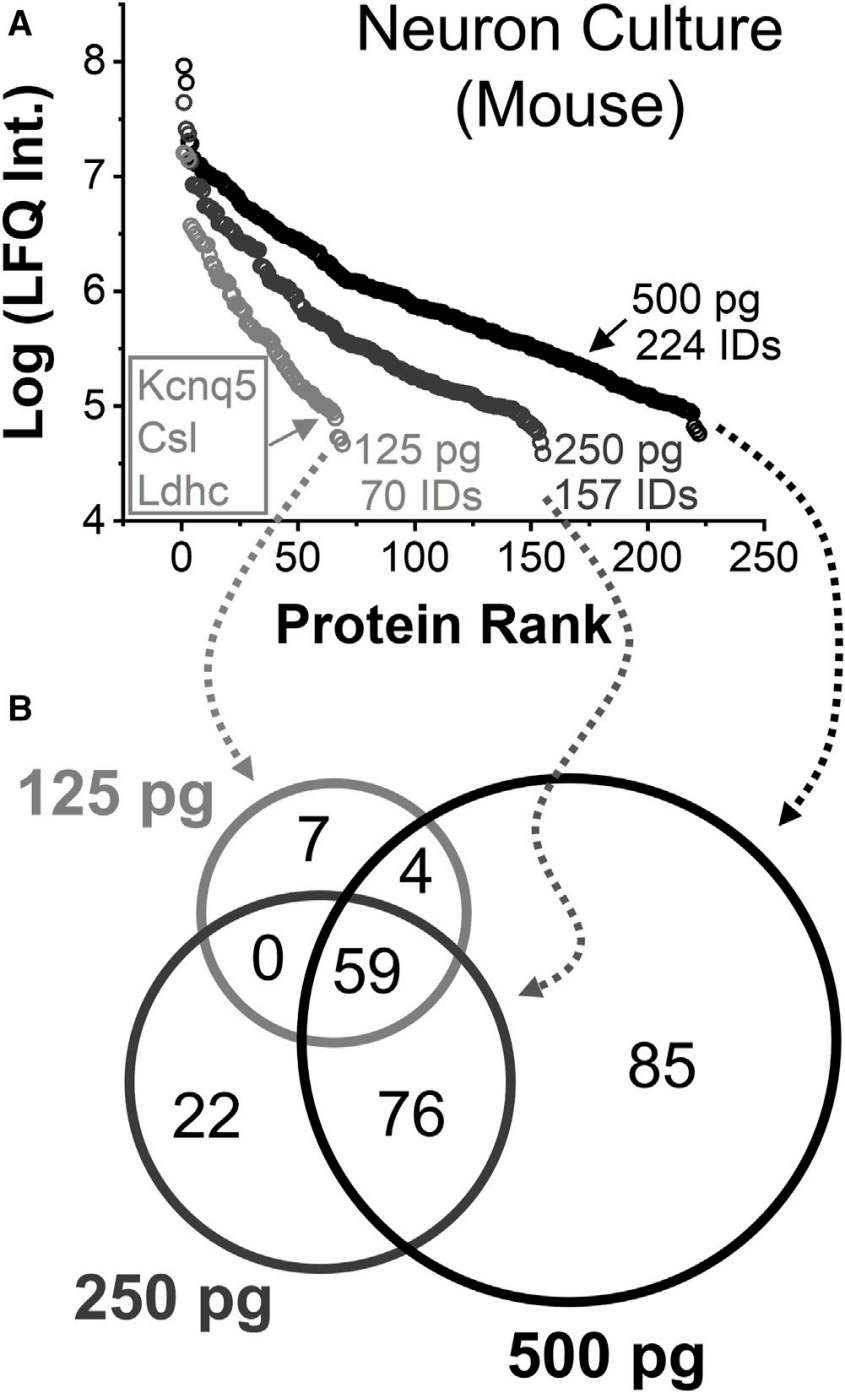
Single-to-subcellular neuroproteomics by CE-HRMS. **(A)** Comparison of the linear dynamic range and depth of the quantified neuroproteome from diluted proteome digests estimating to a single neuron (∼500 pg protein digest), a half neuron (∼250 pg), and quarter of a neuron (∼125 pg). **(B)** Comparison of proteins identified from the diluted digests.

Protein identifications and quantification was assessed. Figure 4A tracks the linear dynamic range of the detected proteins using LFQ as a proxy for concentration. A total of 222 out of 224 identified proteins were quantified from ∼500 pg of protein digest between technical duplicates, approximating to a single neuron. These proteins were found to span over a 3–4 log-order concentration range based on their LFQ abundances. Technical duplicate of 250 pg protein digest, or ca. half a neuron, returned 157 different proteins with 155 quantified. From approximately a quarter of a cell, or ∼125 pg of protein digest, 69 out of 70 identified proteins were quantified. These protein identifications are listed in Supplementary Table S6. The rich information obtained appreciated the molecular state of neuron- and subneuronal equivalent protein amounts.

Smaller protein amounts returned fewer proteins, surprisingly with complementary coverage. Figure 4B annotates the types of proteins that were quantified between the dilutions. Seven proteins were only detected from a quarter portion of the neuronal proteome. Most occupied the lower range of the dynamic concentration span and are known for biological importance. For example, the protein Kcnq5 takes part in forming a potassium channel, which can affect electrical excitability, (Tzingounis et al., 2010), and the protein Gm10358 was linked to carbohydrate degradation during stress response. (Liang et al., 2020). If abundant proteins masked the detection of these proteins in the previous measurements, concentration-dilution-caused enhancements in field-amplified sample stacking or DDA stochasticity may explain the observed signal enhancements.

**FIGURE 5:**
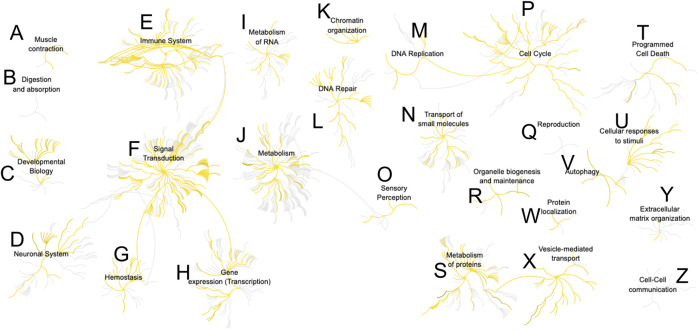
Reactome analysis of protein-protein interactions from a single-neuron equivalent protein digest (500 pg proteome). More than 700 pathways were represented (Supplementary Table S7). Detected nodes are highlighted in yellow. Pathway key: **(A)**, muscle contraction; **(B)**, digestion and absorption; **(C)**, developmental biology; **(D)**, neuronal system; **(E)**, immune system; **(F)**, signal transduction; **(G)**, hemostasis; **(H)**, gene expression; **(I)**, metabolism of RNA; **(J)**, metabolism; **(K)**, chromatin organization; **(L)**, DNA repair; **(M)**, DNA replication; **(N)**, transport of small molecules; **(O)**, sensory perception; **(P)**, cell cycle; **(Q)**, reproduction; **(R)**, organelle biogenesis and maintenance; **(S)**, metabolism of proteins; **(T)**, programmed cell death; **(U)**, cellular responses to stimuli; **(V)**, autophagy; **(W)**, protein localization; **(X)**, vesicle-mediated transport; **(Y)**, extracellular matrix organization; **(Z)**, cell-cell communication.

Last, we sought canonical knowledge on the identified proteins. The 224 proteins (500 pg) were mapped to the human genome and their protein-protein associations analyzed. Figure 5 presents the association network based on Reactome (Jassal et al., 2020). About 750 pathways were represented in the analysis. Multiple cellular functions were accounted for, from cell adhesion, to intracellular signaling, regulation of process outgrowth and synaptic function in neurons. A complete list of the pathways is summarized in Supplementary Table S7. Among the 10 most significantly enriched cellular pathways (*p* ≤ 10^−12^) were Rho GTPase signaling, JAK-STAT signaling, and axon guidance and cell adhesion. Proteins involved in synaptic vesicle release, endocytosis, and synaptic transmission were also enriched. Two processes of interest with a significant P value were also aggrephagy and selective autophagy. Accumulation of misfolded proteins in neurons was associated with several neurodegenerative diseases, such as Parkinson, Alzheimer, and Huntington Disease, (Conway et al., 2020), and the ability to recognize specific targets could identify cellular differences. Detection of a host of important processes with single- and subcellular sensitivity open exciting new directions in neuroscience studies.

## Conclusion

This study found CE to complement nanoLC ESI-HRMS proteomics with sensitivity, speed, and scalability to single cells spanning broad dimensions. From 20-to-100-times less protein digest amounts, CE-ESI was able to identify a similar number of (∼1,100 different) proteins from single identified *X. laevis* cells. The platform was scalable to ∼722 proteins from ∼5 ng of embryo protein digest, supporting extension of the method to smaller cells. Capillary microsampling allowed us to individually aspirate 3 single neurons from culture, identifying 37 different proteins in total. CE-HRMS on neuronal proteome digests estimating to a single neuron (∼500 pg proteome) returned 224 different proteins. The method was tested scalable to 157 proteins from approximately half a neuron (∼250 pg proteome digest) and 70 proteins from about a quarter of a neuron (∼125 pg proteome digest). The quantified proteins were enriched in multiple biological pathways important for cell and nervous system development and cell-to-cell communication. Results from this study demonstrated CE-HRMS-based single-cell proteomics to be scalable for cells measuring ∼35–350 µm in diameter, a ∼150-fold change in available protein amounts, opening new possibilities in cell biology and neuroscience.

These protein identifications mark only the beginning. Further sensitivity enhancements are possible through better sample processing and smarter data acquisition. Micro- and nano-scale platforms that are capable of minimizing protein or peptide losses during sample handling, such as µPOTS (Xu et al., 2019) and nanoPOTS, (Zhu et al., 2018), hold keys to deepen the detectable coverage of the single-cell proteome. Although this work utilized a custom-built ultrasensitive CE-ESI platform, commercialization of instruments capable of low-volume sample handling recently enabled ultrasensitive detection (Johnson et al., 2022) to support adaptation of our approach in other laboratories. CE-ESI-HRMS proteomics opens new possibilities to help characterize the molecules in the cell, now also including the proteome.

## Data Availability

The original contributions presented in the study are publicly available. This data can be found here: http://www.proteomexchange.org/, PXD031955.
